# 

**Published:** 2019-08

**Authors:** Fimka Tozija, Shaban Memeti, Anica Milenkovska

**Affiliations:** ^*a*^Republic of North Macedonia Institute of Public Health, Faculty of Medicine, Republic of North Macedonia.; ^*b*^Republic of North Macedonia Clinic of Traumatology, Republic of North Macedonia.

## Abstract

**Background:**

The main objective of the study is to estimate the burden of road traffic injuries (RTI) in Republic of Macedonia and to suggest preventive measures for policy intervention.

**Methods:**

The study is designed as a retrospective cross-sectional study which analyzes data for RTI for 2015. Data from Ministry of Internal affairs, State Statistical Office, medical records for RTI and WHO HFA DB have been used. WHO standard method and software application have been applied to estimate the burden of road traffic injuries, calculating YLLs, YLDs and DALYs caused by RTI.

**Results:**

The calculation of the burden of road traffic injuries in Macedonia has been done, emphasizing their significance and complexity as a priority public health problem. The estimated burden of RTI on national level is 4 960 DALYs, out of which 3 134 are years of life lost due to premature death (YLLs) and 1 826 years of life lost due to disability (YLDs). The burden in males is three times higher than in females, ie 3 537 disability adjusted life years (71.31%). The most vulnerable age group is from 15 to 29 years, with 1 380 DALYs or 6.1 DALYs per 1 000. Speeding is the most common cause for RTI (31.46%).

**Conclusions:**

The set hypotheses have been confirmed: RTI are a serious public health problem in Macedonia and there is no significant difference between Macedonia and Europe in participation of RTIs in the total burden of disease and injury (coefficient of determination r2 = 0.41875, correlation coefficient (r) = -0.39322 and p = 0.6599; there is a significant difference in the burden of RTI between males and females (χ2 = 28, df = 1, p < 0,01) and it is the highest in the 15-29 years age group, with most of the DALYs lost. Republic of Macedonia has a National strategy for road traffic safety 2015-2020 and comprehensive laws which address most risk factors of RTI, but still their enforcement should be strengthened. Essential cooperation for coordinated multi-sectoral and multidisciplinary preventive activities of all relevant institutions is needed to implement the National strategy as a platform of the Decade of Action for Road Safety 2011-2020.

**Keywords:**

Road traffic injuries, Burden, Mortality, Prevention, Decade of Action

